# Physical Activity and Executive Functions in Adolescents: The Mediating Role of Sleepiness

**DOI:** 10.3390/ijerph191912972

**Published:** 2022-10-10

**Authors:** Fenghua Sun, Fan Zhang, Karen Ying-Fung Ho, Borui Zhang, Zixin Wang, Andy Choi-Yeung Tse

**Affiliations:** 1 Department of Health and Physical Education, The Education University of Hong Kong, Hong Kong SAR, China; 2 Department of Public Health and Preventive Medicine, School of Medicine, Jinan University, Guangzhou 510632, China; 3 Centre for Health Behaviours Research, JC School of Public Health and Primary Care, The Chinese University of Hong Kong, Hong Kong SAR, China

**Keywords:** adolescents, physical activity, cognitive functions, sleepiness

## Abstract

(1) Background: Both physical activity and sleepiness were found to influence the development of executive functioning. The present study aimed to address the effects of different levels of physical activity on adolescents’ executive performance (i.e., working memory and inhibition), and the role of sleepiness in this relationship. It was hypothesized that a higher level of physical activity would be associated with better executive functioning, while this relationship was mediated (at least partially) by reduced sleepiness at school. (2) Methods: 212 adolescents aged from 10 to 17 were recruited, and they were requested to wear accelerometers for seven consecutive days to measure daily levels of physical activity. The Cleveland Adolescent Sleepiness questionnaire (CASQ) was used to assess sleepiness. The working memory and inhibition control were assessed to indicate executive functioning. (3) Results: It was found that sedentary activity was negatively associated with working memory performance, while light and moderate-to-vigorous physical activities were related to better working memory. The relationship between different levels of physical activity and working memory was mediated by reduced sleepiness at school. (4) Conclusions: Our findings provide nuanced evidence that the benefits of light and moderate-to-vigorous physical activity on cognitive development could be explained by reduced sleepiness at school.

## 1. Introduction

Physical activity (PA) refers to a variety of skeletal and muscular movements that lead to energy expenditure. Substantial research shows that PA has an array of benefits for physical, mental, and cognitive health in children and adolescents [1]. Compared with the previous generation, the young generation of this digital age has become less physically active and more sedentary [2]. Generally, the greatest decline in PA across a lifetime occurs during early adolescence [3], which is also a critical period of time for cognitive development [4]. A previous study showed that habitual and recreational PA was associated with better cognitive functions among adolescents [5]. In line with this, a recent systematic review revealed that most previous studies showed that PA was associated with better cognitive functions and academic performance in adolescents [6]. More specifically, higher levels of moderate-to-vigorous physical activity (MVPA) and lower levels of sedentary activity were associated with better reading fluency and comprehension among primary school boys [7]. Although PA appears to be beneficial to adolescents’ cognitive functions, inconsistent findings have remained in the literature [6,8]. In particular, the evidence on the effects and mechanisms of different types of PA (i.e., sedentary, light, moderate, and vigorous PA) has remained limited. It is important to understand the mechanisms in order to design effective PA-based interventions to promote cognitive development in adolescents.

To look into the potential mechanism, the present study investigated the mediating role of sleepiness. The previous literature on sleep has shown that PA is associated with sleep efficiency, sleep duration, and sleep quality [9,10,11]. For example, Lang and colleagues found that higher PA levels predicted better sleep quality [12], whereas van Dyk et al. showed that sedentary activity had a negative association with sleep duration among adolescents [13]. Meanwhile, the existing literature has found that good sleep is a critical contributor to better health, in terms of reduced hypertension risk [14], better mental health [15], and well-developed brain structure [16]. As an important indicator of poor sleep and fatigue, daytime sleepiness has become a common problem among adolescents [17,18,19]. Daytime sleepiness is defined as “the reduced ability to stay awake and alert during normal daytime hours, resulting in lapses of sleepiness or sleep” [20,21]. With increasing electronic media use and academic workloads, 70% of adolescents sleep less than 6 h at least one night per week [22], and approximately one-third have experienced excessive daytime sleepiness, with this prevalence increasing with age [23]. Daytime sleepiness was found to be associated with poorer executive function [24], and sleep deprivation was associated with decreased alertness, attention, and memory, which in turn negatively affects academic performance and mental health [25,26,27]. One recent review has suggested that sleep may help explain how PA affects cognitive functions, while the role of sleepiness in this effect has remained unclear.

Although many studies have examined the relationship between PA, sleep, and cognitive functions in adolescents, very few have looked at the role of sleepiness. Sleepiness is defined as “a great desire to sleep or difficulty in maintaining awakens” [19]. Recent studies on PA and sleepiness have revealed that greater PA may reduce daytime sleepiness [28,29]. Given the previous findings, we suspected that sleepiness might also serve as a potential mediator in the association between PA and cognitive functions. However, to the best of our knowledge, none of the previous studies have investigated this possible mediating role of sleepiness. Therefore, the objectives of the present study are twofold. First, to examine how different levels of PA influenced adolescents’ inhibition control and working memory. Second, to investigate the possible mediating role of sleepiness in this relationship. The findings would address the effect and mechanism of PA on adolescents’ cognitive development, and more importantly, provide critical insights for developing effective PA-based interventions to enhance adolescents’ cognitive functions.

## 2. Materials and Methods

### 2.1. Participants and Study Design

A cross-sectional design was used in the present study to investigate the relationships between PA, sleepiness, and cognitive functions among adolescents in Hong Kong. A total of 212 participants (50% male) from 5th grade (*n* = 105, aged 10–12 years) and 7th grade (*n* = 107, age 13–17 years) were recruited from local public schools using a convenience sampling method. Students who suffered from neurological diseases, color blindness, and with special education needs were excluded from the study. Written consent was obtained from the participants’ parents or guardians before data collection. Ethical approval was obtained from the Human Research Ethics Committee of the Education University of Hong Kong.

### 2.2. Procedures

The data were collected by research staff and student helpers in two sessions separated by a 1-week interval. In the first session, demographic information and participants’ height and weight were measured. Physical fitness parameters such as body composition (measured by an electronic body composition analyzer, TBF-531, Tanita scale, Tokyo, Japan) and maximal oxygen consumption (VO_2max_, measured by the 15-m multi-stage fitness test, MSFT [30]) were also measured. Accelerometers (Actigraph GT3X, ActiGraph LLC., Pensacola, FL, USA) were distributed to measure participants’ physical activity levels in the following 7 consecutive days. Participants were also asked to fill in the Cleveland Adolescent Sleepiness questionnaire (CASQ). In the second session, participants completed the tests of inhibition control and working memory and returned their accelerometers.

### 2.3. Measurements

***Physical activity***. Participants were instructed to wear the accelerometer on the non-dominant wrist for 7 consecutive days (including 5 weekdays and 2 weekend days), and the data were processed by Acti-life software (version 6, ActiGraph LLC., Pensacola, FL, USA). Wearing times of <480 min per day and <4 days (including at least a Saturday or Sunday) in total were considered invalid records. According to Chandler and colleagues [31], <305 vector magnitude counts/5 s are categorized as sedentary behavior, 306–817 counts/5 s are considered as light PA, and >818 counts/5 s are MVPA. The first day’s data were excluded to eliminate the possible novelty effect. In addition, with the above criteria, the percentage of time spent at sedentary activity, LPA, and MVPA during the week was obtained to indicate participants’ different types of PA.

***Sleepiness.*** CASQ was used to measure sleepiness in adolescents [32]. The questionnaire contains 16 items which are categorized into four subscales: (i) sleepiness at school; (ii) alertness at school; (iii) sleepiness on transportation; (iv) sleepiness during the evening. Given the research interest in daytime sleepiness, only subscales of sleepiness at school and sleepiness on transportation were used. All the items are on a 5-point Likert scale ranging from 1 (never) to 5 (almost every day). A higher score implies a higher level of sleepiness. According to one previous study [33], the CASQ has shown good reliability and validity among Chinese adolescents.

***Executive function***. The executive function of the participants was examined using the Eriksen flanker test and Sternberg test from the Cognitive Test Battery which has been widely used in previous studies [34,35].

The Eriksen flanker test was used to measure the participants’ inhibition control. Participants were required to press a button corresponding to the direction of the arrow in the middle and ignore the other four arrows. In congruent trials, the central arrow would point in the same direction as the other four arrows, while in incongruent trials, the central arrow would point in the opposite direction. In total, the task consisted of a practice session with 6 trials and a test session with 60 trials in random order. Participants’ completion time of the task was recorded as reaction time (RT), and the proportion of correct responses was recorded as accuracy (ACC).

The Sternberg test was conducted to test working memory. Participants would first be given sufficient time to memorize a target (e.g., numbers or letters). In the following sessions, whenever the memorized target showed up, participants would press “→”, otherwise, they would press “←”. At the beginning of each part, there were 6 practice trials, and the system would provide “correct” or “incorrect” feedback in these trials to ensure the participants understood the instructions. Each part consisted of 16 trials. The total RT and ACC were recorded to evaluate the participants’ working memory.

### 2.4. Statistical Analysis

The data were analyzed by IBM^®^ SPSS^®^ Statistics 24 (IBM, Armonk, NY, USA). A mediation model (PROCESS model 4) was conducted to analyze the direct and indirect effect of PA on executive function performance [36]. Specifically, the association between PA and cognitive function performance was the total effect (path C), which consisted of a direct effect of PA (path C*) and an indirect effect through sleepiness. The association between PA and sleepiness was path A, and the association between sleepiness and executive performance was path B. Different types of PA, including sedentary activities, light PA, and MVPA were tested separately. The significance of mediating effects was assessed on the basis of the bias-correction bootstrapped 95% confidence intervals [37].

## 3. Results

### 3.1. Descriptive Results

The demographics of the 212 participants (50% male) are shown in Table 1. After screening, the data of 183 participants (*n* = 87 boys and 96 girls) were included in the analysis. Results showed that 80.85% of PA was sedentary activity, while 17.70% was light PA, and 1.45% was MVPA. A gender comparison showed that boys spent more time on MVPA than girls (*p* = 0.005). Moreover, compared with the fifth graders, seventh graders showed greater engagement in sedentary activity, and less in light PA or MVPA. Regarding daytime sleepiness, there was no significant sex difference in the total CASQ score, while the total CASQ score and the scores of the subscales of fifth graders (32.74 ± 5.57) were lower than that of the seventh graders (40.47 ± 8.96, *p* < 0.001).

### 3.2. Associations between PA, Sleepiness, and Working Memory

To test the effect of PA on working memory, participants’ Sternberg test scores were regressed on different types of PA, with sleepiness entered as a mediator (Table 2). It was found that more sedentary activity was associated with higher levels of sleepiness at school (B = 0.039, se = 0.001, t = 4.568, *p* < 0.001) and sleepiness on transportation (B = 0.023, se = 0.008, t = 2.746, *p* = 0.007). Meanwhile, for light PA and MVPA, greater engagement was associated with lower sleepiness at school (for light PA: B = -0.045, se = 0.009, t = −4.715, *p* < 0.001; for MVPA: B = −0.151, se = 0.057, t = -2.648, *p* < 0.001) and on transportation (for light PA: B = −0.023, se = 0.009, t = −2.540, *p* < 0.012; for MVPA: B = −0.161, se = 0.047, t = −3.440, *p* < 0.001). No significant relationship was found between different levels of PA and ACC in the Sternberg test (assessing working memory) after controlling for sex and reaction time.

When testing the mediation models of different levels of PA using the bootstrapping method, it showed that after including sleepiness at school in the model, no direct effect of PA was found. Meanwhile, more engagement in sedentary behavior led to higher sleepiness at school, which was further associated with reduced working memory performance. On the contrary, more engagement in light PA or MVPA was found to be associated with better working memory performance via preventing sleepiness at school. The mediating role of sleepiness on transportation was not significant. To sum up, the effects of different levels of PA on Sternberg test performance were fully mediated by sleepiness at school.

### 3.3. Associations between PA, Sleepiness, and Inhibition

A similar analysis was conducted on the flanker test score. It was found that sedentary behaviour tended to be associated with greater accuracy in the flanker test (B = 0.049, se = 0.025, t = 1.936, *p* = 0.055), while light PA tended to be associated with lower accuracy (B = −0.054, se = 0.028, t = −1.927, *p* = 0.056). No significant effect was found between MVPA and flanker test performance. When testing the mediating effect of sleepiness, no significant direct or indirect effect was found.

## 4. Discussion

The present study aimed to investigate the relationships between different levels of physical activity (i.e., sedentary activity, light PA, MVPA) and executive functions among fifth- and seventh-grade students, and to examine the mediating role of daytime sleepiness in this relationship. It was found that more time in sedentary activity was negatively associated with working memory performance, while more time in light PA and MVPA showed the opposite association. In addition, the relationship between PA and working memory was shown to be mediated by sleepiness at school. The findings advanced our understanding of the effects of PA and sleepiness on executive functions in adolescence, particularly working memory, and provided supporting evidence for promoting PA for young adolescents.

Indeed, it is important to understand not only the relationship between adolescents’ daily PA and cognitive functions but also the underlying mechanism. Among different domains of cognitive functions, the benefits of PA were mainly found in executive functions [6]. A higher level of PA was positively associated with better information processing and executive functions [38], which are rapidly developed in adolescence. It is possible that PA may benefit attention and processing speed, which are the prerequisites for executive functions to be developed. A previous review has suggested that compared with sedentary activities, more intense PA contributes to the development of brain structures and brain functions, thus resulting in better cognitive functions in children [39]. Additionally, PA has been suggested to contribute to the development of the prefrontal cortex [40], where higher cognitive functions such as executive function are located [41]. Meanwhile, PA could also induce growth factor cascades, in terms of increasing brain-derived neurotropic factors, insulin-like growth factor-1, and vascular endothelial growth factor, which contribute to better executive functions and brain development [42]. In addition, longer sedentary time may suggest lower social engagement or an inactive lifestyle, which may have chronic adverse effects on adolescents’ cognitive development. It is also noteworthy that boys engaged more in MVPA, which could contribute to better working memory. Boys’ greater engagement in PA has been widely reported in the previous literature [43,44]. Compared with girls, more boys have met the recommendations for PA (i.e., a minimum of 60 min per day in MVPA), which may be linked to the sex differences in quality of life [44], mental health [45], and cognitive functions [46]. Therefore, our findings have supported that promoting engagement in PA among adolescents, particularly teenage girls, would possibly contribute to better brain development and academic performance.

In the present study, the mediating role of sleepiness at school has extended our understanding of the role of different aspects of sleep in the relationship between PA and executive functions. In a previous study [47], sleep efficiency, but not sleeping duration, served as a mediator in the relationship between PA and executive control among younger and older adults. Despite the merits of objective measures of sleep, the relationship between perceived sleep quality may have a stronger relationship with cognitive functions and PA, which needs to be addressed by further studies. Indeed, among Canadian [48], Japanese [29], and Brazilian adolescents [28], it was found that a higher level of PA was associated with excessive daytime sleepiness among adolescents, which has provided supporting prerequisites for the current mediation model. In our study, the seventh-grade students reported a higher level of sleepiness, which may be related to their lower level of light PA or MVPA. Indeed, higher sleepiness at school was found to mediate the relationship between PA and adolescents’ working memory, but not inhibition control. In line with this, a previous study has shown that adolescents with chronic fatigue showed the most significant impairment in working memory or processing speed [49,50], but not in inhibition. However, given that working memory refers to the storage and manipulation of the information that is necessary for learning, the results may help explain why learning deficits could be found among adolescents with excessive sleepiness or chronic fatigue [18].

The current study extended our understanding of the relationships between different types of PA and executive functions among adolescents and the mediating role of daytime sleepiness in such a relationship. Nevertheless, several limitations need to be acknowledged. First, the causal relationship between PA and cognitive functioning has remained a question. Studies with longitudinal designs or interventions are warranted to explore the causal effects of PA or sleepiness on cognitive development. Second, although objective measurements were adopted for PA and cognitive performance, for sleepiness, only self-reported data were collected to indicate perceived fatigue. Objective measurements for other aspects of sleep, e.g., sleep duration and sleep efficiency, should be included, to further explore the role of sleep in PA and adolescents’ cognition. Third, we only examined the mediating role of sleepiness in the relationship between PA and inhibition control or working memory. However, various cognitive functions, including perception, attention, learning, decision-making, and language abilities, should also be considered in future studies. In addition, more attention should be paid to the effects of sedentary activity. The present study showed that greater engagement in sedentary activity seems to be associated with better accuracy of inhibition control. The findings suggested that sedentary activity may benefit certain cognitive functions. A previous study found that non-screen-based sedentary behavior showed a favorable effect on prospective academic performance among students aged 9–11 compared with MVPA [8]. It is also possible that time spent in sedentary activity could reflect a longer study duration or better concentration. Either way suggests that other potential mediators should be considered in this relationship.

## 5. Conclusions

With a cross-sectional study on students from fifth grade and seventh grade, the current study has found that there was a positive relationship between light PA, MVPA, and working memory performance, while the relationship with sedentary activity was negative. Moreover, adolescents’ perceived sleepiness at school was found to mediate the abovementioned relationships. The findings have extended the existing literature on PA, sleep, and executive functions, as well as provided critical insights for developing PA-based interventions to promote adolescents’ cognitive development and academic performance.

## Figures and Tables

**Table 1:** Descriptive results of participants by sex and school (*n* = 212).

Variables	Overall	Boys	Girls	*p* Value	Secondary School	Primary School	*p* Value
Age	12.47 ± 1.76	12.32 ± 1.76	12.62 ± 1.76	0.213	14.11 ± 0.68	10.80 ± 0.49	<0.001 ***
Height (cm)	154.72 ± 11.46	155.47 ± 14.13	153.98 ± 7.94	0.344	163.48 ± 7.20	145.80 ± 7.36	<0.001 ***
Weight (kg)	45.92 ± 12.8	45.75 ± 14.02	46.09 ± 11.51	0.874	54.06 ± 10.63	37.63 ± 8.93	<0.001 ***
VO_2_max (ml/kg/min)	42.49 ± 9.70	46.37 ±10.70	38.60 ± 6.64	<0.001 ***	46.43 ± 10.18	38.46 ± 7.27	<0.001 ***
Body fat (%)	21.35 ± 6.89	19.08 ± 5.79	23.62 ± 7.18	<0.001 ***	21.97 ± 7.08	20.72 ± 6.67	0.109
Accelerometer (Boy *n* = 87, Girl *n* = 96) (7th grade, *n* = 99; 5th grade, *n* = 84)							
Daily percentage of sedentary behavior ^a^	80.85 ± 7.06	79.88 ± 8.18	81.72 ± 5.77	0.083	84.97 ± 4.42	75.99 ± 6.49	<0.001 ***
Daily percentage of light PA ^a^	17.70 ± 6.23	18.41 ± 7.17	17.06 ± 5.20	0.152	14.12 ± 4.03	21.93 ± 5.70	<0.001 ***
Daily percentage of MVPA ^a^	1.45 ± 1.18	1.71 ± 1.43	1.21 ± 0.83	0.005 **	0.91 ± 0.69	2.08 ± 1.32	<0.001 ***
CASQ (boys *n* = 102, girls *n* = 104) (7th grade, *n* = 102; 5th grade, *n* = 104)							
CASQ total score	36.57 ± 8.38	36.62 ± 9.33	36.52 ± 7.37	0.933	40.47 ± 8.96	32.74 ± 5.57	<0.001 ***
Sleepiness on campus	8.42 ± 4.18	8.86 ± 4.94	7.99 ± 3.24	0.137	10.63 ± 4.62	6.25 ± 2.09	<0.001 ***
Alertness on campus	14.90 ± 3.20	15.05 ± 3.27	14.74 ± 3.13	0.476	15.38 ± 3.29	14.42 ± 3.04	0.031 *
Sleepiness in evening	7.19 ± 2.31	6.96 ± 2.32	7.42 ± 2.28	0.151	7.75 ± 2.08	6.64 ± 2.39	<0.001 ***
Sleepiness during transport	6.05 ± 2.33	5.74 ± 2.26	6.37 ± 2.36	0.052	6.71 ± 2.47	5.41 ± 1.20	<0.001 ***
Executive Function test (7th grade, *n* = 107; 5th grade, *n* = 105)							
Sternberg test accuracy ^b^	96.06 ± 3.15	95.47 ± 3.35	96.64 ± 2.83	<0.001 ***	95.26 ± 3.48	96.90 ± 2.51	<0.001 ***
Sternberg test reaction time (seconds)	770.09 ± 172.27	782.86 ± 171.21	757.68 ± 173.21	0.007 **	674.20 ± 124.42	866.90 ± 159.58	<0.001 ***
Eriksen flanker accuracy ^b^	98.27 ± 2.00	97.84 ± 2.29	98.69 ± 1.58	0.002 **	97.94 ± 2.31	98.61 ± 1.58	0.015 *
Eriksen flanker test reaction time (seconds)	598.53 ± 146.47	605.22 ± 145.57	591.79 ± 147.75	0.507	502.84 ± 76.93	697.00 ± 135.43	<0.001 ***

* *p* < 0.05; ** *p* < 0.01; *** *p* < 0.001. PA = physical activity; MVPA = moderate-to-vigorous physical activity. ^a^ according to Chandler’s cut-off; ^b^ how many correct responses were counted, with 100 as full marks.

**Table 2:** Regression analyses examining the mediating effects of sleepiness on the association between physical activity and Sternberg test performance.

*Baron and Kenny’s 4-Step Approach*	B (95% CI)	*p*-Value
**Step 1 (Path C)**		
Association between PA and Sternberg test accuracy *		
(DV: score of accuracy in Sternberg test ^#^)		
IV: Sedentary activity	−0.002 (−0.071, 0.067)	0.949
LPA	0.007 (−0.069, 0.084)	0.853
MVPA	−0.129 (−0.541, 0.284)	0.540
**Step 2 (Path A)**		
*Association between PA and sleep*		
(DV: sleepiness at school)		
Sedentary activity	0.039 (0.022, 0.056)	<0.001 ***
LPA	−0.044 (−0.063, −0.026)	<0.001 ***
MVPA	−0.152 (−0.265, −0.039)	0.009 **
(DV: sleepiness on transportation)		
Sedentary activity	0.023 (0.006, 0.039)	0.007 **
LPA	−0.024 (−0.042, −0.003)	0.012 **
MVPA	−0.161 (−0.253, −0.068)	<0.001 ***
**Step 3 (Path B)**		
*DV: score of accuracy in Sternberg test, controlling for percentage of Sedentary behavior*		
Sleepiness at school	−0.893 (−1.566, −0.219)	0.010 **
Sleepiness on transportation	0.494 (−0.215, 1.203)	0.171
*DV: score of accuracy in Sternberg test, controlling for percentage of LPA*		
Sleepiness at school	−0.890 (−1.565, −0.215)	0.010 **
Sleepiness on transportation	0.497 (−0.211, 1.204)	0.167
*DV: score of accuracy in Sternberg test, controlling for percentage of MVPA*		
Sleepiness at school	−0.882 (−1.554, −0.210)	0.010 **
Sleepiness on transportation	0.482 (−0.231, 1.196)	0.183
Step 4 (Path C*)		
*Association between cognitive performance Sternberg ^#^ and PA with adjustment for sleep*		
(DV: Cog ^#^; IV: PA)		
Sedentary activity	0.012 (−0.057, 0.081)	0.731
LPA	−0.011 (−0.089, 0.066)	0.772
MVPA	−0.115 (−0.543, 0.313)	0.597
*Total indirect effect of PA on Cog ^#^ through sleep*		
(DV: accuracy in Sternberg test ^#^; IV: sedentary behavior)		
Mediator: sleepiness at school	−0.035 (−0.067, −0.012) *	
Mediator: sleepiness on transportation	0.011 (−0.002, 0.036)	
(DV: accuracy in Sternberg test ^#^; IV: LPA)		
Mediator: sleepiness at school	0.040 (0.014, 0.076) *	
Mediator: sleepiness on transportation	−0.012 (−0.041, 0.001)	
(DV: accuracy in Sternberg test ^#^; IV: MVPA		
Mediator: sleepiness at school	0.134 (0.029, 0.318) *	
Mediator: sleepiness on transportation	−0.078 (−0.242, 0.016)	

# Scores of accuracy in the Sternberg test were adjusted for sex and reaction time; DV: dependent variable of the underlying regression model; IV: independent variable of the underlying regression model; B: regression coefficient; CI: confidence interval; LPA: light physical activity; MVPA: moderate-to-vigorous physical activity. * *p* < 0.05, ** *p* < 0.01, *** *p* < 0.001.

## Data Availability

Not applicable.
